# Early Inflatable penile prosthesis implantation offers superior outcomes compared to delayed insertion following ischemic priapism: a narrative review

**DOI:** 10.1038/s41443-024-00900-y

**Published:** 2024-05-08

**Authors:** Elia Abou Chawareb, Muhammed A. M. Hammad, David W. Barham, Supanut Lumbiganon, Babak K. Azad, Daniar Osmonov, Faysal A. Yafi

**Affiliations:** 1 Department of Urology, University of California, Irvine, CA USA; 2 Department of Urology, Brooke Army Medical Center, Fort Sam Houston, Houston, TX USA; 3 Department of Surgery, Faculty of Medicine, Khon Kaen University, Khon Kaen, Thailand; 4 Department of Urology, University Medical Center Schleswig Holstein Campus Lübeck, Lübeck, Germany

**Keywords:** Erectile dysfunction, Surgery

## Abstract

Ischemic priapism is a urological emergency which may lead to irreversible erectile dysfunction. One of the accepted treatments is penile prosthesis implantation. Given the scarcity of studies directly comparing timing of penile prosthesis insertion after ischemic priapism, consensus remains elusive. We aim to compare different studies in the literature concerning advantages and disadvantages of early versus delayed inflatable penile prosthesis following ischemic priapism. We analyzed 8 articles that investigated immediate and delayed inflatable penile prosthesis placement after ischemic priapism. Early inflatable penile prosthesis placement is associated with better outcomes, including pain relief, priapism resolution, penile shortening prevention, and quicker sexual activity resumption. However, it still carries a high risk of complications like edema, infection, and distal perforations. Delayed inflatable penile prosthesis insertion poses surgical challenges due to the potential for extensive corporal fibrosis. Comparative analyses have shown elevated complication rates in patients with ischemic priapism who undergo delayed inflatable penile prosthesis insertion, as opposed to those with early insertion. In studies reporting complications rates, the total complication rate in the early group was 3.37%, significantly lower than the delayed group (37.23%). Most studies support the superiority of early inflatable penile prosthesis placement following ischemic priapism over delayed placement. Further research is, however, needed to establish a global consensus on timing of prosthesis insertion.

## Introduction

Priapism is characterized by an extended and often painful state of full or partial penile erection lasting beyond four hours [1]. Importantly, this persistent erection remains unrelieved by ejaculation and is unrelated to any form of sexual stimulation [1]. The majority of priapism cases fall into the category of ischemic priapism, characterized by a low-flow state [2]. This condition is considered a urological emergency and is notably linked to irreversible erectile function loss [3]. Ischemic priapism is conceptualized as a compartment syndrome specific to the penis [4]. Refractory ischemic priapism, when left untreated, causes extensive necrosis and complete necrosis of the corporal tissue within 12 and 48 h from the ischemic priapism event, respectively [1]. Ischemic priapism causes time-dependent changes in the metabolic environment within the cavernosal tissue, ultimately leading to smooth muscle necrosis and its substitution with fibrotic tissue [3]. The underlying causes of ischemic priapism predominantly fall into the following categories: hematological or thrombotic triggers [5], pharmaceutical drugs or pharmacological agents [6], intracorporeal injection of pharmaco-stimulants [6], neurological factors [7], and malignancies [8]. However, the aforementioned factors are potential causes, yet in many cases, no specific cause can be identified. The most common cause of priapism in children is sickle cell disease while in adults it is pharmacological agents [6].

One established and effective therapeutic approach for managing ischemic priapism involves the insertion of a penile prosthesis (PP). According to the American Urological Association (AUA), PP placement can be considered in patients with untreated acute ischemic priapism greater than 36 h or in those who are refractory to shunting, with or without tunneling [9]. There are two primary types of PP available: the malleable penile prosthesis and the inflatable penile prosthesis (IPP). Given the infrequent occurrence of priapism and the limited availability of studies directly comparing the timing of PP, and especially IPP, insertion after ischemic priapism, a consensus remains unreachable in this regard. Delayed PP insertion presents a surgical challenge characterized by a high risk of complications [10]. This challenge primarily stems from the development of fibrosis within the corporal tissue where the cylinders are intended to be placed, consequently elevating the complexity of their insertion [11]. Furthermore, prolonged ischemic priapism and the associated fibrosis can contribute to penile shortening and a more challenging-to-treat erectile dysfunction (ED) [12]. Therefore, some surgeons opt for early PP insertion due to its several advantages [12]. This procedure is relatively straightforward for most urologists, effectively alleviates pain, and promptly resolves the priapism episode. Additionally, it plays a crucial role in preserving penile length, preventing further shortening due to corporal fibrosis, and facilitating an earlier return to sexual activity [12]. In this review article, our objective is to comprehensively compare existing studies in the literature that explore the advantages and disadvantages of early versus delayed IPP insertion following ischemic priapism, while focusing on success rates and potential complications.

## Materials and methods

We conducted a detailed literature search utilizing the PubMed and Scopus databases on November 30th, 2023, employing search terms such as “priapism”, “PP”, “penile implant”, and “inflatable penile prosthesis”. The screening process, encompassing titles, abstracts, and full texts, was completed through Covidence (Covidence systematic review software, Veritas Health Innovation, Melbourne, Australia), a web-based software that streamlines systematic review processes by facilitates study screening, data extraction, and team collaboration. Inclusion criteria focused specifically on studies investigating implant insertion after ischemic priapism. Studies not assessing PP after priapism, not written in English, or not assessing patients with IPP were excluded. Two reviewers independently screened the articles, and then collected data from the studies. Data extraction included the timing of the intervention (early vs. delayed), the average duration from ischemic priapism episode to IPP insertion, the number of participants in each group, the most prevalent complications in each group, the satisfaction levels of patients and their partners regarding the IPP, and the study authors’ preference for early or delayed IPP insertion post-ischemic priapism. Our Institutional Review Board (IRB) approval was not needed.

## Results

**Fig. 1:**
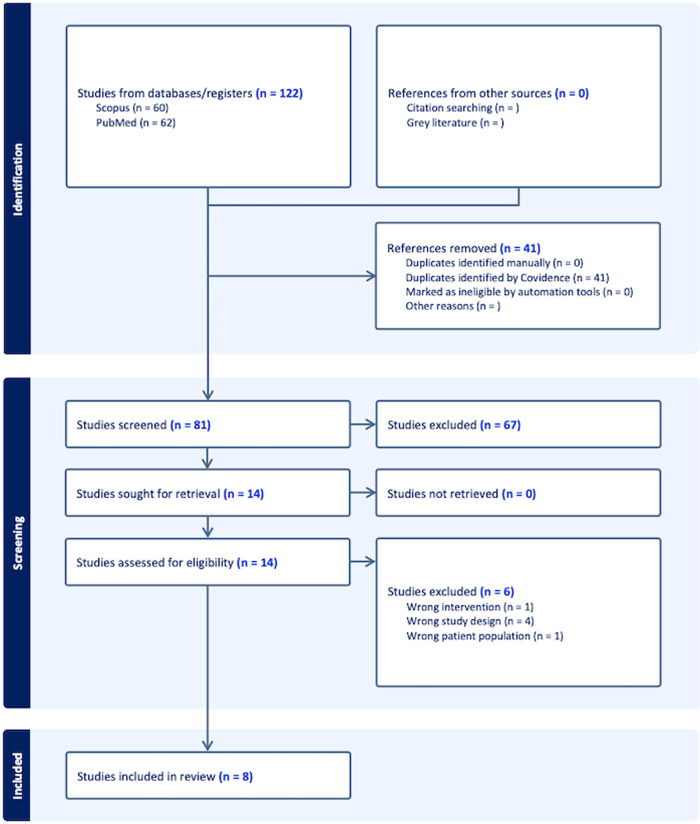
Studies acquisition flowchart.

Of the 122 studies identified, 8 met our criteria, comprising 1 prospective clinical study, and 7 retrospective clinical studies. These selected studies collectively analyze the outcomes and complications associated with early or delayed IPP insertion following ischemic priapism (Fig. 1). Also, some studies were explored to study patient’s satisfaction after the implant insertion. The results are summarized in Table 1.

**Table 1:** Clinical studies focusing on IPP implantation after priapism.

Study	Early or Delayed	Number of Participants	Timing	Complications	Satisfaction	Study’s Preference
Salman et al. [10]	Early	23	1–7 Days	Erosions and Infections	N/A	Early Insertion
	Delayed	19	≥ 3 Months	Corporal Perforation and Urethral Injury	Low	
Sedigh et al. [13]	Early	4	3–20 Days	Hematoma and Penile Sensibility Reduction	High	Early Insertion
	Delayed	3	9–12 Months	Severe Fibrosis and Deformation	N/A	
Ralph et al. [12]	Early	7	1–30 Days	Erosions and Infections	High	Early Insertion
Rees et al. [14]	Early	2	1–8 Days	Deformations	High	Early Insertion
Barham et al. [15]	Early	15	≤ 6 Months	None	High	Early Insertion
	Delayed	47	> 6 Months	Implant Extrusion and Device Cycling Issues	Low	
Zacharakis et al. [4]	Early	3	1–7 Days	Infection and Penile Curvature	High	Early Insertion
	Delayed	15	1–5 Months	Erosions, Infections, and Implant Malfunction	Moderate	

## Discussion

### Timing of PP placement

A significant challenge in the existing literature is the inconsistency in defining the “early” timeframe for the insertion of an IPP following an episode of ischemic priapism. Some studies consider “early” to be within one month or less after the onset of ischemic priapism [4, 10, 12–14], while others extend this period to up to six months post-episode [15]. Research studies have provided evidence indicating that, after six hours of ischemia, various adverse physiological conditions occur within the cavernosa, including anoxia, hypoxia, glucopenia, and acidosis [16, 17]. Anoxia and glucopenia may function as two independent damaging factors to the smooth muscles of the corpora cavernosa [18, 19]. Sedigh et al. conducted early IPP implantation in four patients who had experienced ischemic priapism and had not responded to conservative treatments or shunt surgery. The author’s findings indicated that early placement led to the recovery of sexual function and prevented penile shortening, a common issue in delayed insertions due to fibrotic changes in the corpora cavernosa. During the early implantations, surgeons noted that the prostheses were easily implanted, and the pathological examination of corporal smooth muscle biopsies revealed necrotic cavernosal smooth muscle. Furthermore, the authors reported no intraoperative complications and no instances of infection related to the penile implants. The only postoperative complication observed was a self-resolving penile hematoma, which typically resolved within two weeks. Additionally, the authors noted that all patients who underwent IPP placement reported a reduction in penile sensibility, which persisted for approximately three months [13]. Several studies have explored the outcomes of early IPP insertion, consistently reporting similar findings. In a study by Barham et al., 62 patients who underwent IPP insertion after ischemic priapism were matched with 62 priapism-free patients. Among them, 15 received early IPP insertion, and no complications were observed in both the early and control groups. However, the delayed group exhibited a 40.5% complication rate, primarily non-infectious cylinder-related issues [15]. Ralph et al. investigated 50 patients undergoing early PP insertion, with 7 receiving IPPs. In the IPP group, there were no intraoperative complications, but one patient developed penile deformity after 12 months, and thus needed a revision surgery [12]. Similarly, Rees et al. studied 8 patients, 2 receiving IPPs, with PP implantation after ischemic priapism, with no intraoperative complications, although one patient developed penile deformity due to fibrosis around one cylinder of the IPP. Furthermore, the authors supported the preference of implanting a malleable PP over an IPP, especially in cases where patient compliance with the regular cycling regimen cannot be confirmed preoperatively [14]. In Zacharakis et al.‘s study, early PP insertion in 68 men, including 4 IPPs, within a median of 7 days from ischemic priapism onset showed higher complication rates than in priapism-free patients. However, the early group’s complication rate remained lower than that of the delayed group (9% vs. 27%, respectively). The authors highlighted the straightforward surgical procedure, high patient satisfaction, and successful resumption of sexual activity without complications or postoperative infections associated with early IPP insertion. Penile shortening rate in the early group was 3%, while it was 40% in the delayed group. Similarly, revision rate was lower in the early group when compared to the delayed group (9 vs. 27%, respectively) with Infection being the major cause in both groups [4]. Additionally, it is noteworthy that some patients (14% [12] and 17% [14]) who initially opted for a malleable PP later elected to transition to an IPP, a transition that was accomplished successfully. Moreover, when inflatable cylinders are used, the distal erosion rate would be expected to be less [12].

Similar to the varying definitions of early implantation, the literature also presents diverse interpretations of the timing for delayed IPP implantation. A variety of factors may lead to delayed implantation including time to allow shunt healing, patients desire to try other ED therapies, or a delay in referral to experienced prosthetic surgeon. However, there is limited research available on delayed IPP insertions due to the considerable challenges posed by extensive fibrosis of the cavernosa, even for experienced surgeons. Barham et al. conducted a comparative study that evaluated delayed IPP insertion in patients who had experienced ischemic priapism episodes and compared them to a control group of patients without priapism. Their cohort consisted of 47 (76%) patients who underwent placement more than six months following ischemic priapism episode at a median time of 31.5 months. To note, the median duration of ischemic priapism episode was 60 h ranging from 5 to 144 h in the early group and 33 h ranging from 3 to 168 h in the late group. Thus, patients in the early group experienced longer episodes of ischemic priapism, leading to a more aggressive treatment with early PP implantation. Despite this, patients with shorter episodes still developed refractory ED that required PP implants. The authors’ findings revealed a notably higher complication rate of 40.5% among patients with ischemic priapism who underwent delayed IPP placement. This complication rate was significantly higher than that observed in patients without priapism and even higher than that in ischemic priapism patients who had undergone early IPP placement. Also, the higher rate of complication in the delayed group was attributed to the long time delay from ischemic priapism event to implantation that have led to extensive fibrosis, since most of the postoperative complication faced were corporal related as implant extrusion through corporotomy, proximal migration, and distal extrusion or impeding erosion [15].

Several studies compared the outcomes of early and delayed PP insertions following ischemic priapism. The recent AUA guidelines did not reach a consensus on whether early or delayed PP placement is superior. In their guidelines, they emphasize that clinicians should discuss the risks and benefits of both with the patient [9]. As for the European Association of Urology (EAU) guidelines, cases of delayed presentation or when injection therapy and shunting fail, implantation of a PP may be considered, but if a shunt has been performed, implantation should be delayed to reduce infection risk [20]. Diagnostic tools like MRI and smooth muscle biopsy can help assess viability for shunting, and the choice of prosthesis depends on patient suitability, surgeon experience, and equipment availability [20, 21]. Ralph et al. emphasized the challenges associated with delayed PP insertion, particularly the presence of significant corporal fibrosis. This fibrotic tissue often necessitates extended or double corporotomies and the use of specialized cavernotomes during the surgical procedure. Consequently, only downsized and shorter prosthesis cylinders are able to be accommodated in the scarred corporeal bodies in such cases [12]. The reduction in penile length that can occur following ischemic priapism is a significant concern in PP surgery. Deveci et al. emphasized the importance of this issue, noting that lower satisfaction rates and lower scores on the International Index of Erectile Function (IIEF) were reported when penile length was compromised [22]. Zacharakis et al. conducted a study in which they observed distinct differences between the early and delayed implantation groups. In the early implantation group, the tunica albuginea at the site of the corporotomy was noted to be significantly edematous and thickened. However, the PP was successfully implanted without encountering intraoperative complications, and the dilatation of the corpora was generally easy, with only few cases of minor distal fibrosis observed. While in the delayed implantation group, the corporal dilatation proved to be challenging due to the presence of dense fibrosis. This necessitated penile degloving and a second distal corporotomy in a majority of the patients. The study’s findings concluded that delayed IPP implantation in patients with severe corporal fibrosis resulting from ischemic priapism presents a real surgical challenge, and this approach is associated with higher complication rates and lower levels of patient satisfaction [4].

**Fig. 2:**
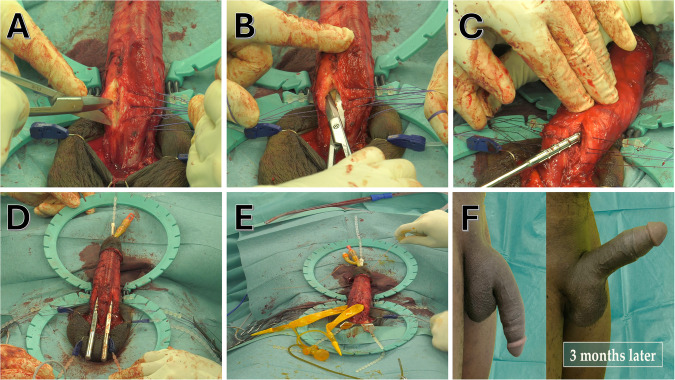
IPP Insertion after priapism. All pictures provided courtesy of Dr. Daniar Osmonov. Used with permission. **A** Corporotomy of the Scarred Tunica Albuginea. **B** Backward Cutting of Right Distal Cavernosa. **C** Dilatation of the Corpora with the Uramix Cavernosome Set®. **D** Dilatation of Bilateral Cavernosomes. **E** Placement of Inflatable Cylinders. **F** Final Results 3 Months Post-operation.

Hebert et al. explored the ideal timing for IPP insertion in cases involving scarred corporal bodies resulting from ischemic priapism or prior PP infections, which comprised the majority of the investigated cases. Their research revealed that the rate of postoperative complications significantly increased when implantation was performed more than four months after the onset of fibrosis. In the delayed ( > 4 months) implantation group, the postoperative complications observed were of a more severe nature, including infection, urethral erosion, and impending glans erosion. As a result of their findings, Hebert et al. concluded that the most favorable timing for IPP implantation is within four months of the inciting event that leads to scarring of the corporal bodies. This timeframe was associated with lower complication rates and better surgical outcomes [11]. Additionally, some authors recommended the consideration of a slight delay in early implantation, waiting for a few weeks after the onset of the priapic episode. This strategic timing window is chosen to avoid the potential complications associated with hematomas and edema, which could contribute to an increased infection rate. Moreover, within this specific time window, significant fibrotic changes in the corpora would not be apparent yet, thus reducing the overall risk of complications [4]. (Table 1 and Fig. 2).

### Patient’s satisfaction

PP implantation as a treatment for ED, regardless of its underlying cause, consistently results in a high level of patient satisfaction [23]. Notably, IPP tend to exhibit an even higher satisfaction rate when compared to malleable ones, all while maintaining comparable complication rates [24, 25]. Simsek et al. conducted a survey in which they discovered that a remarkable 88.9% of patients who had undergone the implantation of a three-part IPP for various causes expressed satisfaction with the procedure. However, the remaining patients reported dissatisfaction, primarily attributable to either post-surgical pain or penile shortening. Moreover, the study revealed an impressive 94.4% partner satisfaction rate, with the majority of patients recommending the implantation procedure [26]. Shah et al. reached the conclusion that achieving the highest satisfaction rate among patients undergoing IPP implantation, for any underlying cause, involves a comprehensive approach. This approach includes preoperative counseling and the establishment of realistic expectations for the patient. Additionally, it includes tailoring device selection and counseling to each patient’s unique condition and comorbidities, paying attention to partner satisfaction, and effectively managing and controlling the rate of postoperative complications [27]. Moreover, Ralph et al. had a patient satisfaction rate of 96% after early PP insertion following an ischemic priapism event [12]. There remains uncertainty regarding whether delaying the implant insertion by up to a week after a priapism episode could potentially alleviate the psychological impact of the surgery, possibly resulting in a higher satisfaction rate. Such a delay may afford patients the necessary time to gain a better understanding of their condition, and simultaneously, the surgical procedure may be less challenging due to a potentially less acute pathological state [13].

The literature reviewed in this study has certain limitations. The available studies exhibit diverse designs, with a limitedness of prospective clinical trials. Additionally, many studies suffer from small sample sizes. The definition of timing as “early” or “delayed” insertion varies across studies, and follow-up durations are inconsistently defined. Given that our analysis comprises nonrandomized and unblinded retrospective studies, coupled with reviews of these studies, evaluating their quality posed a challenge.

## Conclusion

The placement of IPP to address ED in men with a history of ischemic priapism consistently results in a high satisfaction rate. However, it is essential to note that this approach is also associated with a notable incidence of complications, particularly in cases of delayed placement. The majority of studies have demonstrated the superiority of early implantation over delayed implantation. Nonetheless, to establish a global consensus on the most appropriate timing for IPP insertion and to further validate these findings, additional prospective clinical trials are warranted. Finally, it is imperative that patients who have experienced ischemic priapism are promptly referred to a prosthetic urologist to minimize the complications associated with delayed IPP placement.
